# A bibliometric analysis of metastatic breast cancer: two-decade report (2002-2022)

**DOI:** 10.3389/fonc.2023.1229222

**Published:** 2023-08-24

**Authors:** Siyuan Jiang, Qingjie Meng, Fuqing Ji, Yulong Yin, Xianghua Liu, Wenzhen Shi, Yonggang Lyu

**Affiliations:** ^1^ Department of Thyroid Breast Surgery, Xi’an NO.3 Hospital, the Affiliated Hospital of Northwest University, Xi’an, Shaanxi, China; ^2^ Clinical Medical Research Center, the Affiliated Hospital of Northwest University, Xi’an No.3 Hospital, Xi’an, Shaanxi, China; ^3^ Xi’an Key Laboratory of Cardiovascular and Cerebrovascular Diseases, the Affiliated Hospital of Northwest University, Xi’an No.3 Hospital, Xi’an, Shaanxi, China

**Keywords:** bibliometric analysis, metastatic breast cancer, co-word analysis, cocitation analysis, research trends analysis

## Abstract

**Background:**

MBC is a lethal form of breast cancer that arises when cancer cells invade other organs or tissues. The treatment of MBC needs personalized approaches based on the tumor and patient characteristics. The purpose of this paper is to analyze MBC studies from 2002 to 2022 using bibliometrics and to investigate its current situation, main contributors, core journals, highly cited papers, and topic evolution.

**Materials and methods:**

We retrieved data from Web of Science Core Collection (WOSCC). Bibliometric analysis of the included literatures mainly used the following tools: the function of “analyze results” and “citation report” in WoS, Microsoft excel 2021, CiteSpace v.6.1. R6, VOSviewer v.1.6.18, BICOMB v.2.04 and gCLUTO v.1.0.

**Results:**

We found 12,653 articles on MBC research published in 1, 802 journals by 69, 753 authors from 118 countries. The annual output and citation of MBC articles showed a rising trend over time. The United States was the most influential country in MBC research. The most cited journal in this field was The Journal of Clinical Oncology. And the most cited article was by Slamon DJ. The co-word analysis of keywords divides MBC into six research clusters. The hormone receptor-positive MBC and liquid biopsy of MBC are the frontiers research trends. “CDK4/6 inhibitor” had the highest burst strength.

**Conclusion:**

Our bibliometric analysis offers a comprehensive overview of MBC research in the past two decades. It shows the current situation, main contributors, core journals, highly cited papers, and topic evolution of this field. Our study can assist researchers and practitioners to comprehend the development and trends of MBC research and to discover potential directions for future research.

## Introduction

Breast cancer is a health challenge. It is one of the most common cancers, about 11.7% of cancer patients is breast cancer. It also ranks fifth among the deadliest cancers, claiming 685,000 lives annually (1). Despite advances in treatment, many breast cancer patients face a grim prognosis (2). About 30% of them develop metastatic breast cancer (MBC) within 5-20 years after their initial diagnosis (3, 4). This means that the cancer cells have escaped from the original site and invaded other organs or tissues (5). MBC is responsible for 90% of breast cancer deaths, and its 5-year survival rate is only 26% (6, 7). MBC is a heterogeneous disease, requiring personalized treatment based on various factors (8). These include the ages, general conditions, prognostic factors, and the markers, such as estrogen receptor (ER), progesterone receptor (PR), human epidermal growth factor receptor-2 (HER2) and Ki-67 (9). Recently, researchers have been searching for specific therapeutic targets to improve their outcomes. This has become a hot topic in MBC research.

Bibliometrics is a method that uses quantitative statistics to analyze publications. It can explore the output and impact of researchers, institutions, countries, and reveal research trends, frontiers, hotspots, knowledge structure (10). While, no bibliometric analysis of MBC was retrieved. This paper aims to fill this gap by using bibliometric methods to investigate the state of the art, main contributors, core journals, highly cited papers, topic evolution and so on about MBC research from 2002 to 2022. we hope to identify potential future directions and hot issues that deserve attention.

## Materials and methods

### Data and retrieval method

**Figure 1:**
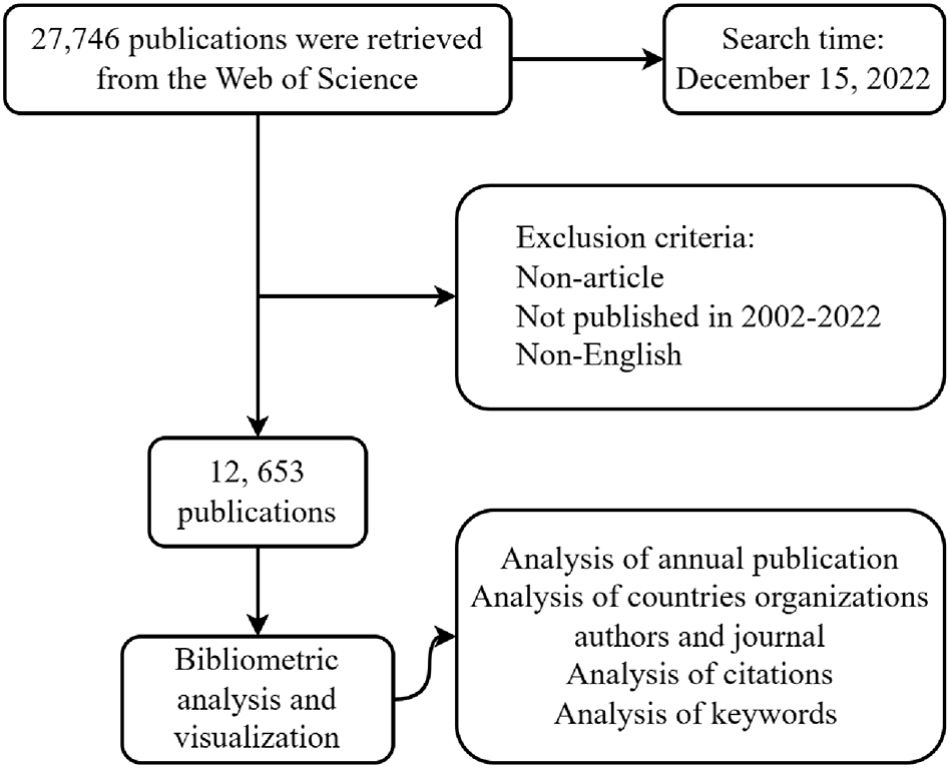
Flowchart.

WOSCC database was employed to conduct a literature search. To ensure consistency, we performed all literature search and data extraction on December 15, 2022. We employed the following search strategies: subject word = “metastatic breast cancer”, document type = article, language = English, publication year = 2002-2022, as depicted in **Figure 1**. We extracted data on title, author, publication year, country/region, institution, keywords, abstracts, references, etc. Our analysis included 12,653 publications. We downloaded all records and citations in TXT format for further analysis (**Supplementary Materials**).

### Data analysis

The bibliometric analysis of the included literatures mainly used the following tools: the function of “analyze results” and “citation report” in WoS, Microsoft excel 2021, CiteSpace v.6.1.R6, VOSviewer v.1.6.18, BICOMB v.2.04 and gCLUTO v.1.0.

The “analyze results” in the WoS was used to obtain the number of publications of different authors, years, country/region, affiliates and journals. Simultaneously, we obtained the number of citations without self-citations (Nc) from the function of “citation report” in WoS, as well as H-Index (11) of different authors, country/regions, affiliates and journals. Moreover, we employed the impact factor (IF) to evaluate the impact of the journal.

Microsoft Excel 2021 charted the number of publications per year in a bar chart and analyzed growth trends from 2002 to 2022.

CiteSpace, a freely available Java-based software, enables the visualization and analysis of trends. It facilitates a comprehension of the structure and development of aa field of study, with particular emphasis on significant turning points and key references (12). We employed CiteSpace for conducting references co-citation and keyword burst analysis. The former allowed us to discern the knowledge structure and the evolution of frontiers. The latter involved the computation of the frequency and growth rate of keywords in different time periods to identify the keywords with sudden dominance, thereby reflecting the explosive growth of a topic or technology in a certain period (13). We selected the following parameters: Years per slice: 1; Selection criteria: g-index (K = 25), link retaining factor (LRF = 3), L/N = 10, e = 1.0; Pruning mode: pathfinder.

VOSviewer is also a bibliometrics visual analysis software tool (14). We used it to visualize the co-authorship network between countries/regions as well as institutions and employed it for carrying out co-occurrence analysis of keywords. Moreover, it was used to obtain the total link strength and average publication year (APY) between different countries/regions, institutions, and keywords.

BICOMB, a software for biomedical text mining, enables the extraction of keywords from bibliographic databases and the construction of co-occurrence matrices. Another software, gCLUTO, is a cross-platform graphical application that facilitates the clustering of datasets with low or high dimensions and the analysis of the attributes of the different clusters. It also provides tools for visualizing the outcomes of the clustering solutions using various plots, such as tree, matrix, and an OpenGL-based mountain plot (15). We imported TXT files into BICOMB to obtain the high frequency keywords. Subsequently, by importing co-word matrix into gCLUTO, we conducted bi-cluster analysis with the aim of identifying the research hotspot. We also generated the visual mountain map and heat map.

## Results

### Analysis of the annual number of publications

**Figure 2:**
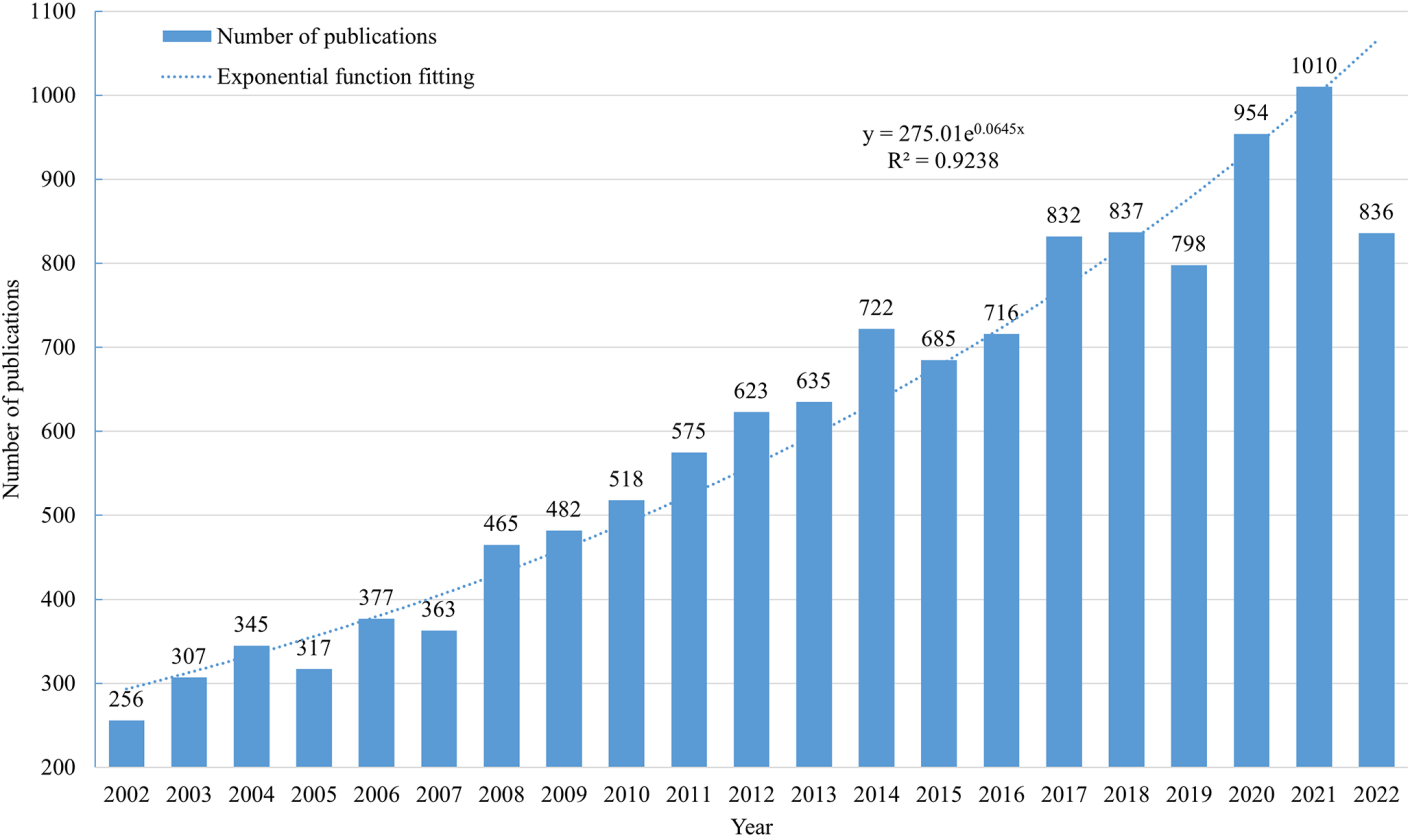
The trend of MBC publications from 2002 to 2022.

From 2002 to December 15, 2022, researchers published 12,653 articles on MBC. Furthermore, the number of articles published exhibited an upward trend over time (**Figure 2**). After fitting the data of the annual number of publications, we found that the annual number of publications in MBC increased exponentially (y=275.01e^0.0645x^, R^2 =^ 0.9238). This suggested that MBC had significant clinical implications and potential for development.

### Analysis of countries/regions and organizations

**Figure 3:**
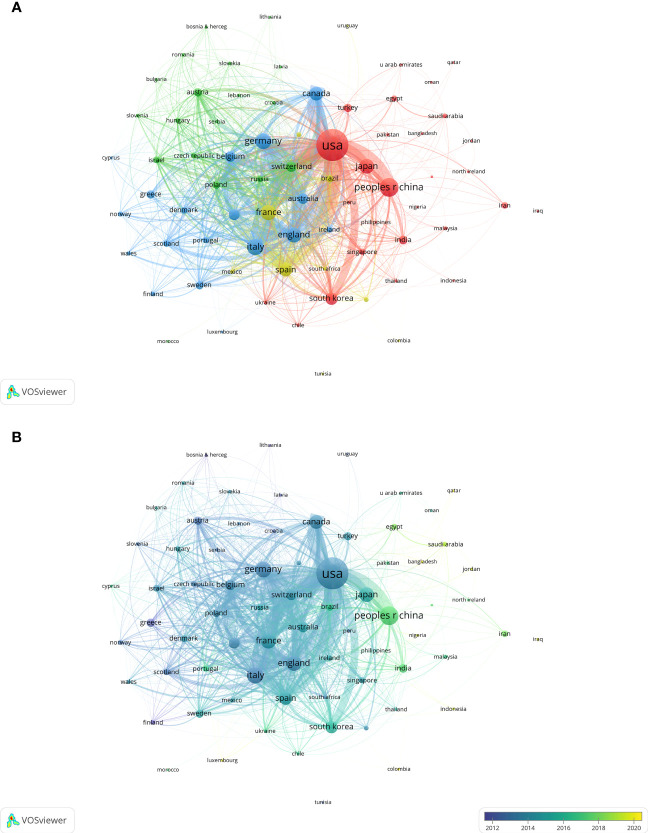
Visualization of key countries/regions for MBC research. **(A)** Visualization of the network of cooperation between countries/regions. **(B)** Visualization of the overlay of cooperation between countries/regions.

The field of MBC had 118 countries/regions as contributors, out of which 74 countries/regions had more than 5 articles each. As shown in **Table 1**, the United States (5,278 articles, 41.71%), China (1,491 articles, 11.78%) and Italy (1,074 articles, 8.49%) were the top three countries with the largest number of publications. However, in the aspect of Nc and H-Index, the top four countries/regions were the United States (Nc=288,674, H-Index=236), England (Nc=70,961, H-Index=133), Germany (Nc=58,904, H-Index=113) and France (Nc=57,953, H-Index=119), which suggested that the United States had the highest productivity and influence in this field. We also visualized the co-authorship analysis of them with at least five documents each using VOSviewer (**Figure 3A**). The node indicated the number of articles, while the line indicated the degree of cooperation. The United States occupied the central position and cooperated with various countries such as France, China, Japan, England, Italy, Spain, etc. While, we also found that the United States (3,976), England (2,168) and France (1,894) had the highest total link strength. Additionally, although China had the second highest number of articles published, its total link strength was only 799. The overlay visualization map in VOSviewer (**Figure 3B**) revealed that some countries such as the United States had an earlier development in this research field with an APY of 2013.65. In contrast, China had a later development with an APY of 2017.31.

**Table 1:** The top 10 countries/regions with the highest number of publications.

Rank	Countries/Regions	Article Counts	Proportion	Nc	H-Index
1	USA	5278	41.71%	288674	236
2	CHINA	1491	11.78%	40592	86
3	ITALY	1074	8.49%	49464	103
4	GERMANY	1004	7.93%	58904	113
5	ENGLAND	909	7.18%	70961	133
6	FRANCE	863	6.82%	57953	119
7	JAPAN	823	6.50%	25037	71
8	CANADA	723	5.71%	44222	100
9	SPAIN	614	4.85%	40299	100
10	SOUTH KOREA	522	4.13%	27961	73

**Figure 4:**
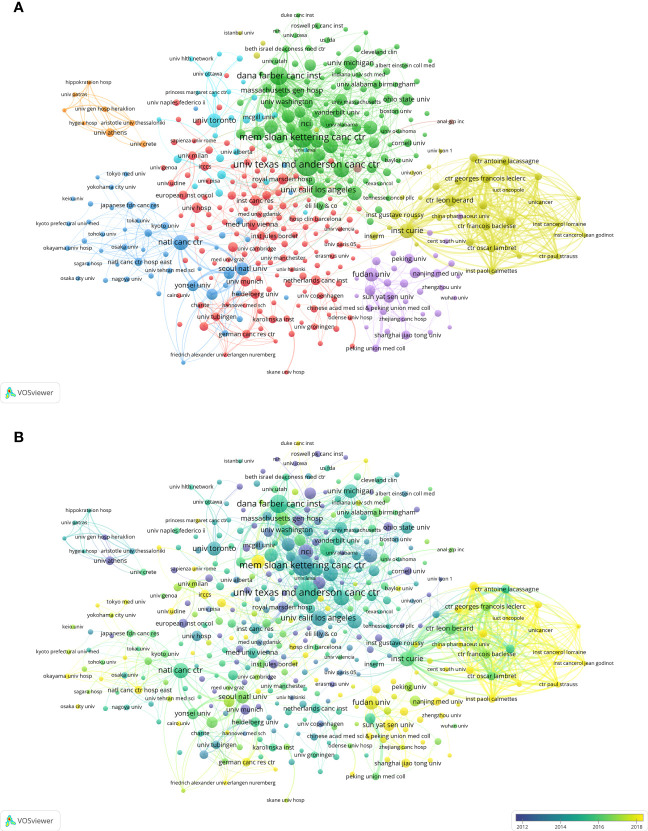
Visualization of key institutions for MBC research. **(A)** Visualization of the network of cooperation between institutions. **(B)** Visualization of the overlay of cooperation between institutions.

The research in this field involved the participation of 13,252 institutions. **Table 2** illustrated that The University of Texas MD Anderson Cancer Center (391 articles), Memorial Sloan Kettering Cancer Center (335 articles) and Dana-Farber Cancer Institute (256 articles) occupied the first three positions in terms of the quantity of articles published. However, Memorial Sloan Kettering Cancer Center (Nc=33,859, H-Index=86), Dana-Farber Cancer Institute (Nc=24,565, H-Index=80) and The University of Texas MD Anderson Cancer Center (Nc=21,334, H-Index=78) emerged as the leading three institutions in terms of Nc and H-Index. Furthermore, we employed VOSviewer to generate a visual representation of the co-authorship patterns among institutions that had at least 20 documents each (**Figure 4A**). The clusters exhibited strong internal connections, but weaker inter-cluster relations. The institutions with the highest total link strength were Dana-Farber Cancer Institute (1,170 articles), Memorial Sloan Kettering Cancer Center (1,141 articles) and Institut Curie (1,114 articles). Based on the overlay visualization map of VOSviewer, we observed that French and Chinese institutions entered this area of research relatively late, and that French institutions collaborated more closely with each other (**Figure 4B**).

**Table 2:** The top 10 organizations with the highest number of publications.

Rank	Organizations	Article Counts	Proportion	Nc	H-Index
1	The University of Texas MD Anderson Cancer Center	391	3.09%	21334	78
2	Memorial Sloan Kettering Cancer Center	335	2.65%	33859	86
3	Dana-Farber Cancer Institute	256	2.02%	24565	80
4	University of California, San Francisco	189	1.49%	14863	53
5	University of California, Los Angeles	185	1.46%	16799	63
6	National Cancer Center	184	1.45%	11607	47
7	Institut Curie	179	1.41%	12411	55
8	National Cancer Institute	177	1.40%	12919	61
9	University of Pittsburgh	176	1.39%	8024	51
10	Genentech Inc.	170	1.34%	21962	64

### Analysis of journals and authors

As shown in **Table 3**, the field had witnessed the publication of articles by 1,802 journals, of which 225 journals contributed more than 10 articles each. The Breast Cancer Research and Treatment (602 articles), Clinical Cancer Research (342 articles) and Journal of Clinical Oncology (293 articles) were the top three journals with the highest quantity of publications. However, in terms of Nc and H-Index, which measure the citation impact and research quality respectively, Journal of Clinical Oncology attained the first position with an Nc of 56,351 and an H-Index of 131, while Clinical Cancer Research (Nc=30,700, H-Index=98) and Annals of Oncology (Nc=18,281, H-Index=76) secured the second and third positions respectively.

**Table 3:** The top 10 journals with the highest number of publications.

Rank	Journal	Article Counts	Proportion	Nc	H-Index	IF (2021)
1	Breast Cancer Research and Treatment	602	4.76%	17251	64	4.624
2	Clinical Cancer Research	342	2.70%	30700	98	13.801
3	Journal of Clinical Oncology	293	2.32%	56351	131	50.739
4	Annals of Oncology	286	2.26%	18281	76	51.769
5	Clinical Breast Cancer	250	1.98%	4719	31	3.078
6	Breast	235	1.86%	4313	34	4.254
7	BMC Cancer	229	1.81%	6060	41	4.638
8	PLOS ONE	210	1.66%	7260	44	3.752
9	Anticancer Research	206	1.63%	3381	31	2.435
10	British Journal of Cancer	205	1.62%	9503	55	9.082

The field of MBC research encompassed 69,753 authors, who varied in their publication output and impact. We found that Andreas Schneeweiss (95 articles), Javier Cortés Castán (89 articles) and Hope S. Rugo (87 articles) were the three authors with the highest number of articles, respectively. However, the authors with the highest Nc, which indicates citation impact, were José Baselga Torres (Nc=11,768), Javier Cortés Castán (Nc=10,908) and Mario Campone (Nc=8,412). Furthermore, the authors with the highest H-Index, which reflects both quantity and quality of publications, were Klaus Pantel (H-Index=39), José Baselga Torres (H-Index=39) and Javier Cortés Castán (H-Index=36) (**Table 4**).

**Table 4:** The top 10 authors with the most publications and Nc.

Rank	Author	Article Counts	H-Index	Author	Nc	H-Index
1	Andreas Schneeweiss	95	32	José Baselga Torres	11768	39
2	Javier Cortés Castán	89	36	Javier Cortés Castán	10908	36
3	Hope S. Rugo	87	32	Mario Campone	8412	24
4	Massimo Cristofanilli	81	35	Massimo Cristofanilli	8191	35
5	Gabriel N. Hortobagyi	78	33	Klaus Pantel	7827	39
6	Klaus Pantel	77	39	Seock-Ah Im	7664	30
7	Seock-Ah Im	74	30	Sung-Bae Kim	6982	23
8	Binghe Xu	74	20	Edith A. Perez	6703	30
9	Nadia Harbeck	69	24	Andreas Schneeweiss	6653	32
10	Eric P. Winer	66	30	Tadeusz Pienkowski	6228	17

### Analysis of co-cited references

**Figure 5:**
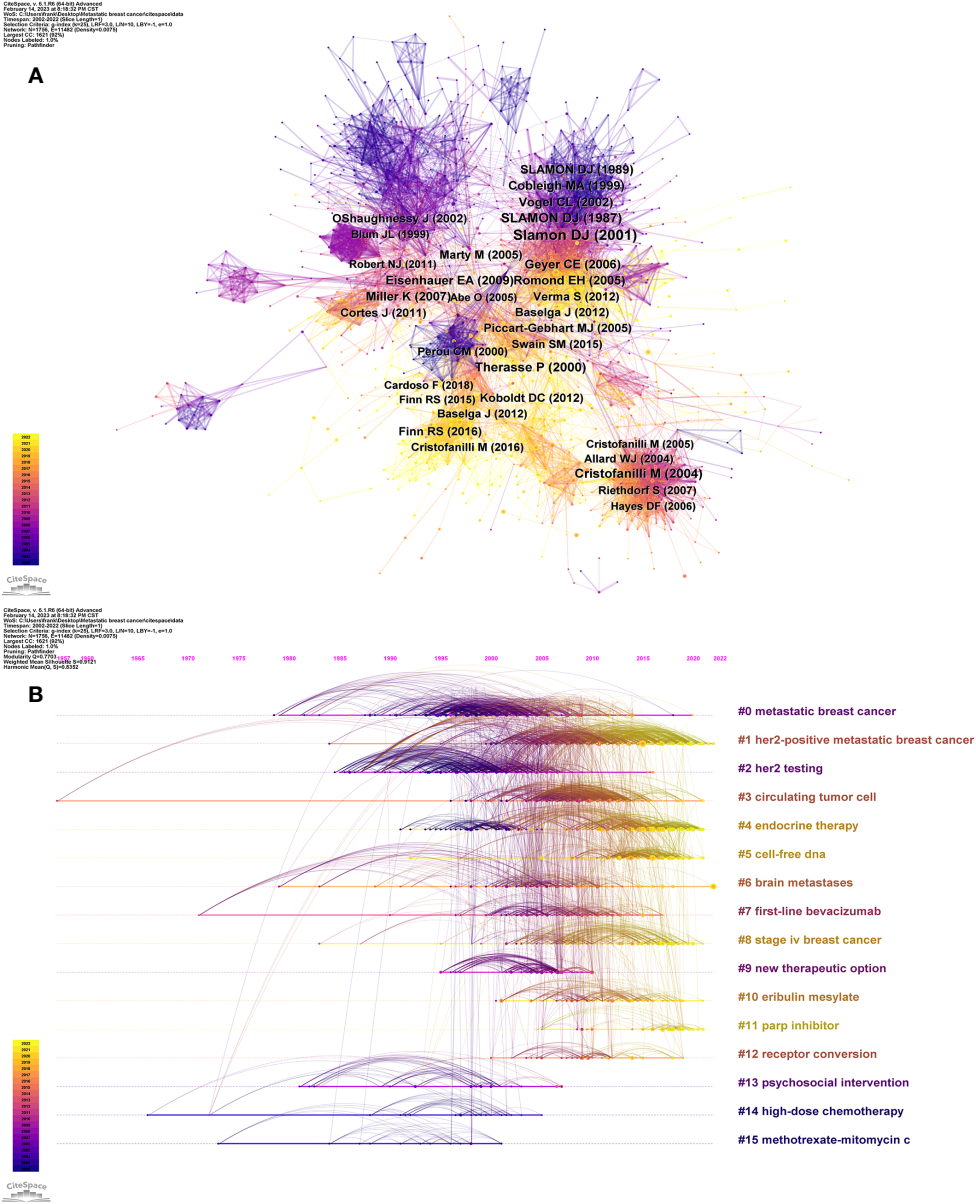
Visualization of reference co-citation network and time line. **(A)** Reference co-citation network. **(B)** Reference timeline view.

To examine the core literature, important topics, and development trends and trajectories of MBC field, we performed a co-citation analysis of literature using CiteSpace. **Figure 5A** presented a visual representation of the co-cited references, comprising 1,756 nodes and 11,482 links. **Table 5** summarized the top ten references in citation frequency and centrality in this field. The publication with the highest citation frequency was by Slamon DJ (2001) (n = 1,483). While, the second and the third were the Slamon DJ (1987) (n = 760) and Cristofanilli M (2004) (n = 665). The publications with higher centrality, which indicated the influence of a publication in the network, were by Greenberg PAC (1996) (centrality = 0.12), followed by Slamon DJ (1989) (centrality = 0.07) and Riethdorf S (2010) (centrality = 0.07).

**Table 5:** The top 10 co-cited references based on citation counts and centrality.

Rank	Citation Counts	References	DOI	Centrality	References	DOI
1	1483	Slamon DJ, 2001, NEW ENGL J MED, V344, P783	10.1056/NEJM200103153441101	0.12	Greenberg PAC, 1996, J CLIN ONCOL, V14, P2197	10.1200/JCO.1996.14.8.2197
2	760	Slamon DJ, 1987, SCIENCE, V235, P177	10.1126/science.3798106	0.07	Slamon DJ, 1989, SCIENCE, V244, P707	10.1126/science.2470152
3	665	Cristofanilli M, 2004, NEW ENGL J MED, V351, P781	10.1056/NEJMoa040766	0.07	Riethdorf S, 2010, CLIN CANCER RES, V16, P2634	10.1158/1078-0432.CCR-09-2042
4	608	Therasse P, 2000, J NATL CANCER I, V92, P205	10.1093/jnci/92.3.205	0.06	Cristofanilli M, 2004, NEW ENGL J MED, V351, P781	10.1056/NEJMoa040766
5	506	Eisenhauer EA, 2009, EUR J CANCER, V45, P228	10.1016/j.ejca.2008.10.026	0.06	Johnston S, 2009, J CLIN ONCOL, V27, P5538	10.1200/JCO.2009.23.3734
6	492	Vogel CL, 2002, J CLIN ONCOL, V20, P719	10.1200/JCO.2002.20.3.719	0.06	Kaufman B, 2009, J CLIN ONCOL, V27, P5529	10.1200/JCO.2008.20.6847
7	489	Cobleigh MA, 1999, J CLIN ONCOL, V17, P2639	10.1200/JCO.1999.17.9.2639	0.05	Slamon DJ, 1987, SCIENCE, V235, P177	10.1126/science.3798106
8	466	Geyer CE, 2006, NEW ENGL J MED, V355, P2733	10.1056/NEJMoa064320	0.05	Miller K, 2007, NEW ENGL J MED, V357, P2666	10.1056/NEJMoa072113
9	424	Miller K, 2007, NEW ENGL J MED, V357, P2666	10.1056/NEJMoa072113	0.05	Sledge GW, 2003, J CLIN ONCOL, V21, P588	10.1200/JCO.2003.08.013
10	413	Slamon DJ, 1989, SCIENCE, V244, P707	10.1126/science.2470152	0.05	Fossati R, 1998, J CLIN ONCOL, V16, P3439	10.1200/JCO.1998.16.10.3439

Moreover, based on the log-likelihood ratio (LLR) algorithm, we performed a clustering analysis of co-cited references, that extracted cluster labels based on the most salient terms in each cluster. The analysis yielded 16 clusters, which encompassed various topics such as metastatic breast cancer (cluster #0), her2-positive metastatic breast cancer (cluster #1), her2 testing (cluster #2), circulating tumor cell (cluster #3), endocrine therapy (cluster #4), cell-free DNA (cluster #5), brain metastases (cluster #6), first-line bevacizumab (cluster #7), stage iv breast cancer (cluster #8), new therapeutic option (cluster #9), eribulin mesylate (cluster #10), parp inhibitor (cluster #11), receptor conversion (cluster #12), psychosocial intervention (cluster #13), high-dose chemotherapy (cluster #14) and methotrexate-mitomycin c (cluster #15). The clusters had average silhouette values above 0.8, which signified reliable and significant clustering quality. **Figure 5B** depicts the timeline of different clusters, showing the active time of different clustering topics of co-cited references. **Table 6** provided the details of the 16 clusters.

**Table 6:** Top 16 largest clusters of co-cited references.

Cluster ID	Size	Silhouette	Mean (year)	Top terms (log-likelihood ration, p-level)
0	282	0.872	2000	metastatic breast cancer (33893, 1.0E-4); phase ii study (26053.84, 1.0E-4); first-line chemotherapy (25660.09, 1.0E-4); weekly docetaxel (20912.61, 1.0E-4); her2-positive metastatic breast cancer (17395.53, 1.0E-4)
1	223	0.852	2011	her2-positive metastatic breast cancer (82757.77, 1.0E-4); trastuzumab emtansine (78550.48, 1.0E-4); her2-positive breast cancer (21337.09, 1.0E-4); human epidermal growth factor receptor (19711.43, 1.0E-4); 2-positive metastatic breast cancer (17307.24, 1.0E-4)
2	178	0.922	2000	her2 testing (8047.09, 1.0E-4); trastuzumab therapy (5688.99, 1.0E-4); her2-overexpressing metastatic breast cancer (5498.52, 1.0E-4); trastuzumab-based treatment (5382.05, 1.0E-4); trastuzumab emtansine (5040.84, 1.0E-4)
3	177	0.957	2008	circulating tumor cell (132909.22, 1.0E-4); circulating tumour cell (11263.83, 1.0E-4); molecular characterization (8257.72, 1.0E-4); peripheral blood (6694.55, 1.0E-4); small-cell lung cancer (5965.34, 1.0E-4)
4	176	0.895	2011	endocrine therapy (42273.75, 1.0E-4); hormone receptor-positive (27059.57, 1.0E-4); cyclin-dependent kinase (21029.84, 1.0E-4); postmenopausal women (19147.25, 1.0E-4); aromatase inhibitor (18871.73, 1.0E-4)
5	108	0.907	2015	cell-free dna (17995.34, 1.0E-4); circulating tumor dna (7739.3, 1.0E-4); circulating cell-free dna (4809.94, 1.0E-4); circulating tumour dna (4683.08, 1.0E-4); estrogen receptor (4315.13, 1.0E-4)
6	107	0.918	2005	brain metastases (18889.44, 1.0E-4); metastatic breast cancer cell (16602.25, 1.0E-4); breast cancer metastasis (14853.17, 1.0E-4); breast cancer cell (8942.98, 1.0E-4); metastatic triple negative breast cancer (8619.06, 1.0E-4)
7	91	0.956	2006	first-line bevacizumab (9329.12, 1.0E-4); antiangiogenic therapy (5458.64, 1.0E-4); metronomic chemotherapy (4612.44, 1.0E-4); her2-negative metastatic breast cancer (3509.24, 1.0E-4); athena study (2973.34, 1.0E-4)
8	81	0.941	2011	stage iv breast cancer (16893.88, 1.0E-4); *de novo* (8648.98, 1.0E-4); recurrent metastatic breast cancer (8360.75, 1.0E-4); population-based study (7316.55, 1.0E-4); locoregional therapy (5554.6, 1.0E-4)
9	44	0.984	2004	new therapeutic option (860.62, 1.0E-4); epothilone b analog ixabepilone (810.67, 1.0E-4); incorporating recent data (635.8, 1.0E-4); clinical perspective (635.8, 1.0E-4); integrating epothilone (635.8, 1.0E-4)
10	43	0.946	2011	eribulin mesylate (21074.96, 1.0E-4); absolute lymphocyte count (6191.24, 1.0E-4); eribulin monotherapy (5156.28, 1.0E-4); eribulin mesilate (5123.9, 1.0E-4); phase ii multicenter (4727.03, 1.0E-4)
11	30	0.96	2016	parp inhibitor (2020.08, 1.0E-4); mutational burden (1747.73, 1.0E-4); asco guideline (1452.97, 1.0E-4); 2 mutation (1433.36, 1.0E-4); high tumor (1328.53, 1.0E-4)
12	29	0.958	2009	receptor conversion (2340.94, 1.0E-4); biomarker discordance (1535.19, 1.0E-4); receptor status (1183.91, 1.0E-4); metastatic relapse (855.84, 1.0E-4); different distant breast cancer metastases (845.45, 1.0E-4)
13	23	0.999	1995	psychosocial intervention (2301.59, 1.0E-4); supportive-expressive group therapy (1976.67, 1.0E-4); cancer patient (1664.48, 1.0E-4); psychosocial support (1180.24, 1.0E-4); psychological intervention (1144.13, 1.0E-4)
14	18	0.997	1994	high-dose chemotherapy (2084.12, 1.0E-4); autologous stem cell transplantation (851.22, 1.0E-4); undergoing high-dose chemotherapy (851.22, 1.0E-4); autologous hematopoietic stem cell transplantation (459.06, 1.0E-4); patient selection (459.06, 1.0E-4)
15	11	0.994	1991	methotrexate-mitomycin c (150.03, 1.0E-4); randomized controlled (150.03, 1.0E-4); cooperative group (117.25, 1.0E-4); metastatic breast carcinoma (109.07, 1.0E-4); first-line single-agent mitoxantrone (78.01, 1.0E-4)

### Analysis of keywords

Keywords are concise and abstract indicators of a specific topic that can provide a general overview of the literature theme by summarizing the literature comprehensively. By using keywords that have high frequency, the research hotspots and other key issues in a discipline can be effectively identified as they illustrate the research hotspots.

**Figure 6:**
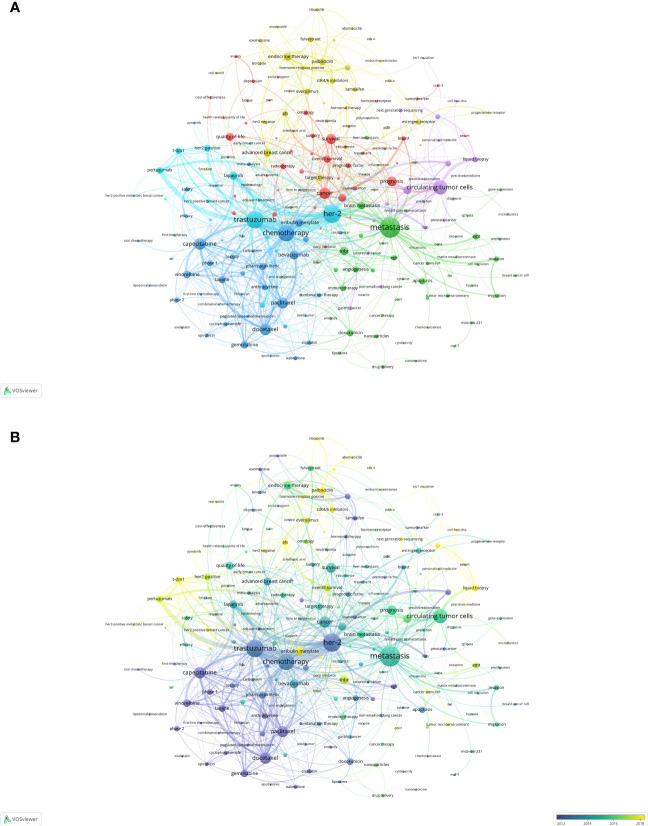
Keyword co-word analysis **(A)** Keyword network visualization. **(B)** Keyword overlay visualization.

We used VOSviewer to merge keywords (**Supplementary Materials**) and also performed author keyword co-occurrence analysis, resulting in more than 14,000 keywords with a frequency exceeding 20 for 213 of them. For the purpose of enhancing visual clarity, we omitted the two keywords “breast cancer” and “metastatic breast cancer” and generated the network and the overlay visualizations. In **Figure 6A**, the 211 keywords were segregated into six clusters denoted by purple, cyan, green, yellow, red and blue respectively. By identifying the semantic connections between the keywords within each cluster, we determined six research themes for MBC research: Cluster 1 (cyan) concentrated on targeted therapy for HER2-positive MBC and its cardiotoxicity; Cluster 2 (blue) concentrated on clinical trials and combination chemotherapy regimens for MBC; Cluster 3 (green) concentrated on investigating molecular mechanisms and novel treatment methods for MBC; Cluster 4 (purple) concentrated on clinical application of liquid biopsy in MBC; Cluster 5 (red) concentrated on diagnosis, comprehensive treatment, prognosis evaluation and quality of life in MBC; Cluster 6 (yellow) concentrated on treatment and resistance mechanisms for hormone receptor-positive MBC. **Figure 6B** displayed an overlay visualization map of the keywords with pyrotinib (APY=2021.044), CDK4(APY=2020.739), CDK4/6 inhibitors (APY=2020.423), abemaciclib (APY=2020.371), PD-L1(APY=2020.087) having higher frequency recently.

**Figure 7:**
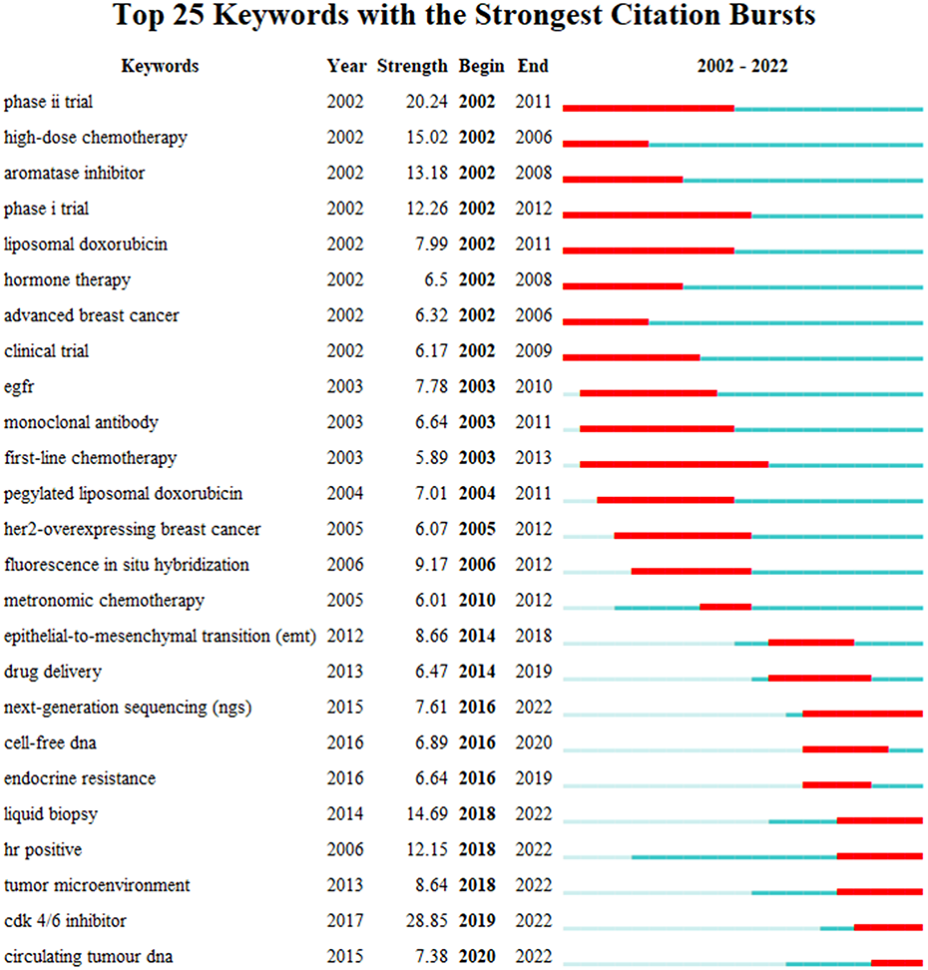
Top 25 keywords with the strongest citation bursts.

To corroborate the robustness of our clustering outcomes, we employed BICOMB and gGLUTO to extract the high-frequency keywords and preform the bi-clustering analysis. We observed that the order of the keywords with frequency of occurrence ranked 68 was equal to their frequency of occurrence. Hence, we designated the keywords with a rank above 68 as the high-frequency keywords (**Table 7**). Utilizing these high-frequency keywords, we devised a word-document matrix (**Supplementary Materials**) and applied gCLUTO for bi-clustering analysis. Subsequently, we generated a mountain map and a heat map from this analysis. The mountain map (**Supplementary Figure 1A**) displayed six discrete and well-defined clusters, each annotated with a cluster number. The magnitude and altitude of each mountain corresponded to the quantity and resemblance of keywords in its cluster. The heat map (**Supplementary Figure 1B**) exhibited a clustering tree of articles comprising high-frequency words. The congruence of representative high-frequency keywords within clusters with those derived from VOSviewer clustering attested to the quality of clustering outcomes. Moreover, we utilized CiteSpace for the analysis of strongly cited keywords with burst. **Figure 7** showed the top 25 most burst keywords, such as “CDK4/6 inhibitor” (28.85), such as the “CDK4/6 inhibitor” (28.85), which was also the most contemporary burst keyword. And the “first-line chemotherapy” (2003-2013) was the keyword with the longest burst. Additionally, recent burst keywords encompassed “HR positive” (12.15), “tumor microenvironment” (8.64) and “circulating tumor DNA” (7.38), which reflected current research hotspots.

**Table 7:** High-frequency Keywords.

Rank	Keywords	Frequency, n	Percentage, %	Cumulative Percentage, %
1	breast cancer	3511	7.5201	7.5201
2	metastatic breast cancer	2145	4.5943	12.1145
3	metastasis	860	1.8420	13.9565
4	her-2	737	1.5786	15.5350
5	trastuzumab	729	1.5614	17.0965
6	chemotherapy	571	1.2230	18.3195
7	circulating tumor cells	506	1.0838	19.4033
8	docetaxel	335	0.7175	20.1208
9	capecitabine	333	0.7132	20.8340
10	paclitaxel	331	0.7090	21.5430
11	cancer	279	0.5976	22.1406
12	survival	251	0.5376	22.6782
13	prognosis	232	0.4969	23.1751
14	bevacizumab	214	0.4584	23.6335
15	lapatinib	193	0.4134	24.0469
16	biomarker	192	0.4112	24.4581
17	quality of life	189	0.4048	24.8629
18	endocrine therapy	184	0.3941	25.2570
19	advanced breast cancer	183	0.3920	25.6490
20	bone metastases	173	0.3705	26.0195
21	brain metastasis	164	0.3513	26.3708
22	gemcitabine	158	0.3384	26.7092
23	vinorelbine	158	0.3384	27.0476
24	t-dm1	156	0.3341	27.3818
25	eribulin	153	0.3277	27.7095
26	her-2 positive	152	0.3256	28.0350
27	taxanes	149	0.3191	28.3542
28	pharmacokinetic	147	0.3149	28.6690
29	overall survival	146	0.3127	28.9818
30	triple negative breast cancer	144	0.3084	29.2902
31	cdk 4/6 inhibitors	138	0.2956	29.5858
32	palbociclib	135	0.2892	29.8749
33	doxorubicin	131	0.2806	30.1555
34	epithelial-mesenchymal transition (EMT)	124	0.2656	30.4211
35	pertuzumab	124	0.2656	30.6867
36	targeted therapy	122	0.2613	30.9480
37	apoptosis	120	0.2570	31.2050
38	everolimus	113	0.2420	31.4471
39	immunohistochemistry	112	0.2399	31.6869
40	angiogenesis	112	0.2399	31.9268
41	estrogen receptor	109	0.2335	32.1603
42	liquid biopsy	108	0.2313	32.3916
43	aromatase inhibitors	106	0.2270	32.6187
44	fulvestrant	104	0.2228	32.8414
45	pfs	103	0.2206	33.0620
46	radiotherapy	103	0.2206	33.2826
47	clinical trial	102	0.2185	33.5011
48	immunotherapy	100	0.2142	33.7153
49	egfr	97	0.2078	33.9231
50	prognostic factors	96	0.2056	34.1287
51	cardiotoxicity	92	0.1971	34.3257
52	hormone receptor positive	90	0.1928	34.5185
53	invasion	89	0.1906	34.7091
54	nab-paclitaxel	88	0.1885	34.8976
55	oncology	87	0.1863	35.0840
56	circulating tumor dna	84	0.1799	35.2639
57	toxicity	83	0.1778	35.4417
58	combination therapy	82	0.1756	35.6173
59	tamoxifen	76	0.1628	35.7801
60	safety	75	0.1606	35.9407
61	her-2 negative	74	0.1585	36.0992
62	vascular endothelial growth factor (VEGF)	73	0.1564	36.2556
63	phase 1	73	0.1564	36.4119
64	cisplatin	70	0.1499	36.5619
65	positron emission tomography (pet)	70	0.1499	36.7118
66	next generation sequencing	70	0.1499	36.8617
67	breast	69	0.1478	37.0095
68	drug resistances	68	0.1456	37.1552

## Discussion

We conducted a bibliometric analysis of publications related to MBC in WOSCC over a period of more than 20 years (2002-2022). We applied various criteria to screen the retrieved records and included a total of 12653 articles in our final sample. We employed several tools and methods, such as “analyze results” and “citation report” in WoS, Microsoft excel 2021, CiteSpace v.6.1.R6, VOSviewer v.1.6.18, BICOMB v.2.04 and gCLUTO v.1.0, to examine the annual distribution, institutional affiliation, authorship, journal outlet, co-citation network, author keywords and other aspects of the publications on MBC. Through this comprehensive analysis, we have initially explored the basic research overview, development trends and possible future research hotspots regarding MBC. As **Figure 2** illustrated, the quantity of publications in the MBC field had grown steadily over the last 20 years. This suggested that this field had received considerable attention and recognition from the scientific community.

We have also carried out a comparative analysis of the countries/regions and institutions covered by the publications about MBC. The results indicated that the United States and its affiliates dominated this area, with its outstanding performance in total number of publications, Nc and link strength, ranking first in the world. Further analysis shows that although China ranks second only to the United States in quantity of publications, it lagged behind England, France, Germany and other countries in terms of Nc and H-Index. **Figure 3B** also demonstrated that China, India and other developing countries entered the MBC research later than USA and other developed countries. These findings suggested that China’s publication output had increased rapidly in recent years, but it still lacked high-level, high-quality research. This implied that latecomers in this field should focus more on producing innovative and rigorous research rather than merely increasing the quantity of literature, and should foster more collaboration with institutions from other countries.

We also evaluated MBC-related journals according to IF, Nc and H-Indices. Annals of Oncology had the highest IF, but its Nc and H-Index were weaker than Journal of Clinical Oncology and Clinical Cancer Research. Journal of Clinical Oncology had the highest Nc and H-Index, indicating that it provided a large number of high-quality and influential researches for this field. Clinical Cancer Research followed closely behind Journal of Clinical Oncology in terms of Nc and H-Index. These data would assist researchers in selecting journals when submitting or retrieving high-quality articles related to MBC.

This study also identified the most prolific and influential authors based on the quantity of publications, Nc and H-Index. Prof. Andreas Schneeweiss ranks top in quantity of publications, followed by Prof. Javier Cortés and Prof. Hope S. Lugo. Professor José Baselga Torres ranked first in Nc with his publications of the interim and final report of the CLEOPATRA trial, which showed the triple combination of Pertuzumab, Trastuzumab and Docetaxel improved the survival in patients with HER2-positive MBC, providing an important basis for subsequent clinical practice and research (16, 17). Javier Cortés Castán, Sung-Bae Kim, Seock-Ah Im and Andreas Schneeweiss also contributed to the CLEOPATRA trial. Professor Klaus Pantel, who had the highest H-Index among the authors, focused on circulating tumor cells (CTCs) and circulating tumor DNA(CtDNA) (18). These findings indicated that these authors had conducted extensive and rigorous research in MBC.

Co-citation analysis measured the degree of association and similarity between documents by counting how often two or more documents were cited together in other documents. It helped identify important documents and authors in a field, as well as explore the topics and knowledge bases among documents (19). **Table 5** showed that the most frequently cited reference in the MBC field was a clinical trial by Dennis J. Slamon entitled “*Use of chemotherapy plus a monoclonal antibody against HER2 for metastatic breast cancer that overexpresses HER2*” and published in The New England Journal of Medicine in 2001 (20). This trial suggested that trastuzumab could significantly improve the prognosis of HER2-positive MBC patients, providing a new targeted therapy option for this group. It also showed the importance and feasibility of personalized treatment based on molecular markers. The second most cited document was still by Dennis J. Slamon. It investigated the correlation of HER2/neu oncogene amplification and expression with breast cancer recurrence (21). These indicated Professor Dennis J. Slamon’s outstanding contribution to targeted therapy for HER2-positive MBC.

In terms of centrality, Greenberg PAC (1996) (22), Slamon et al. (1989) (23) and Riethdorf S (2010) (24) occupied the top three positions respectively. They presented: long-term follow-up results of MBC patients who achieved complete remission (CR) after treatment with doxorubicin and alkylating agents’ chemotherapy regimen; the correlation between amplification of HER-2/neu in human breast cancer cells and recurrence and survival; detection and characterization of CTCs levels in peripheral blood of breast cancer patients before and after neoadjuvant therapy. These constituted landmark documents in distinct research directions in MBC field.

As illustrated in the timeline view of the references (**Figure 5B**), we partitioned them into three stages. Before 2000: MBC was primarily classified and managed according to clinical features and hormone receptor status. Chemotherapy was one of the main treatment methods, but with limited efficacy (25). This stage chiefly employed psychosocial intervention, high-dose chemotherapy, methotrexate-mitomycin c, etc. as reference words. From 2000 to 2010: Breast cancer was beginning to be recognized as a heterogeneous disease. Molecular subtypes determine its different biological features and clinical behavior (26). The detection of HER2 receptor and the emergence of the targeted drug trastuzumab brought new therapeutic alternatives for HER2-positive MBC patients (20). Simultaneously, fulvestrant marked the commencement of a new era in endocrine therapy (27–29), but there was still a lack of effective treatments for triple-negative or basal-like MBC. This stage predominantly used her2 testing, her2-positive metastatic breast cancer, new therapeutic alternative, receptor conversion and other terms as reference words. From 2010 to present: The diagnosis and treatment of MBC has entered a new stage, involving more molecular markers, new drugs and precision medicine. For instance, CTCs and cell-free DNA can be used to monitor tumor burden, predict prognosis and guide treatment choices (30, 31); bevacizumab, PARP inhibitors and other new drugs can provide more options for MBC patients (32–34). This stage is mainly characterized by cell-free DNA, PARP inhibitor, eribulin mesylate and other reference words.

Keyword co-occurrence analysis and cluster analysis identified six research topics in the field of MBC. Cluster 1: Targeted therapy for HER2-positive MBC; Cluster 2: Chemotherapy for MBC; Cluster 3: Molecular mechanisms of MBC; Cluster 4: Liquid biopsy for MBC; Cluster 5: Survival and quality of life of MBC; Cluster 6: Hormone receptor-positive MBC treatment.

### Cluster 1: Targeted therapy for HER2-positive MBC

HER2 acts as a transmembrane tyrosine kinase and is involved in physiological processes related to cell growth, differentiation and survival (35). Under physiological conditions, the HER2 receptor binds to a growth factor and activates downstream signaling pathways (36). However, aberrant amplification or mutation of the gene leads to overexpression or sustained activation of pathways, which is conducive to the growth, aggression and metastasis of tumor cells, and confers resistance to apoptosis and drug treatment (35). The role of HER2 in breast cancer was first elucidated by Slamon et al. in 1987. They examined its amplification in 189 breast cancer tissues and demonstrated that the amplification was significantly correlated with high clinical stage, presence of lymph node metastasis, ER non-expression and worse outcomes. They also found that patients with HER2-amplified breast cancer had good responsiveness to doxorubicin and cyclophosphamide (21). Trastuzumab, the first anti-HER2 drug is that targets the extracellular structural domain of the HER2 receptor, suppresses its dimerization and activation, and causes immune-mediated cytotoxicity (37). Trastuzumab was approved for marketing by the FDA in 1998 and has extended the survival time of patients with this type of breast cancer (38).

Pertuzumab, another anti-HER2 drug, was approved for marketing by the FDA in 2012 (39). The simultaneous use of these two anti-HER2 drugs is known as dual HER2 blockade, which synergistically inhibits the HER2 signaling pathway and enhances anti-tumor activity by blocking the formation of HER2 heterodimers. The combination therapy had been corroborated by the CLEOPATRA trial (16, 40), which suggested that dual-targeted drug use significantly improved PFS and OS in HER2-positive MBC patients. Recent guidelines and expert opinions suggested that the combination of the dual anti-HER2 agent with a chemotherapeutic agent should be considered the first-line standard of care for patients who had relapsed after 6 months of adjuvant therapy (41).

In addition to monoclonal antibodies, a distinct category of minuscule molecule therapeutics can target HER2 receptors, namely tyrosine kinase inhibitors (TKIs). TKIs can infiltrate the cell by oral administration, attach to the intracellular domain of HER2 receptors, and directly inhibit their kinase activity. A number of TKIs have been used to treat such patients, such as lapatinib and neratinib (42, 43). The benefit of TKIs is that they can pass through the blood-brain barrier and therefore have a better effect on patients with brain metastasis from breast cancer (44). In addition, the disadvantage of this class of drugs is that they can cause adverse reactions, such as rash and diarrhea (45).

A novel class of anti-HER2 agents, namely antibody-drug conjugates (ADCs), has garnered considerable interest in recent times. These agents consist of cytotoxic drugs, linkers and monoclonal antibodies, which leverage the high affinity and specificity of antibodies to selectively deliver cytotoxic drugs to tumor cells, then release its to induce tumor cell death, thereby achieving targeted therapy (46). Two ADCs have been developed so far, namely T-DM1 (Trastuzumab emtansine) and T-DXd (Trastuzumab deruxtecan). T-DM1 comprises trastuzumab and DM1 (a microtubule stabilizer), which has been utilized as a second-line or later-line treatment in people who exhibits progression after trastuzumab and pertuzumab treatment (47).

### Cluster 2: Chemotherapy for MBC

The treatment of cancer with drugs that can either eradicate or impede the proliferation of malignant cells is known as chemotherapy. Cytotoxic drugs are commonly used to treat breast cancer. The first drug of this kind, nitrogen mustard, received clinical approval in 1946 (48). In 1975, Dr. Gianni Bonadonna from Italy introduced CMF (cyclophosphamide, methotrexate, fluorouracil) for breast cancer patients who had positive lymph nodes (49). Fifteen years later, it was demonstrated that the efficacy of the AC (doxorubicin/cyclophosphamide) chemotherapy regimen was comparable to that of CMF. Subsequently, in 1998, it was revealed that the combination of AC and paclitaxel had superior efficacy than AC alone. Moreover, in 2003, it was discovered that administering chemotherapy every 2 weeks, rather than every 3 weeks, could result in better outcomes for patients. Furthermore, in 2006, TC4 (docetaxel/cyclophosphamide, 4 cycles) achieved better PFS and OS than AC4 (doxorubicin/cyclophosphamide, 4 cycles). Finally, in 2017, researchers suggested that TC6 was non-inferior to various TaxAC regimens (doxorubicin/cyclophosphamide-paclitaxel) (50).

Although the outcomes of breast cancer patients with specific molecular markers have been significantly extended by targeted drugs and endocrine therapy. However, disease progression and/or treatment resistance were experienced by MBC patients. Therefore, most MBC patients still require monotherapy or combination regimens with cytotoxic chemotherapy. The trastuzumab and chemotherapy exhibit synergistic activity for HER2-positive breast cancer, as paclitaxel, doxorubicin and capecitabine show additional activity in combination with trastuzumab (51). For MBC patients with endocrine resistance or extensive symptomatic visceral involvement, chemotherapy can still benefit them (52). For triple-negative MBC patients, monotherapy chemotherapy has typically been employed as initial treatment, but combination chemotherapy has been recommended for rapidly progressive visceral disease (50). Moreover, a search for clinical trials of targeted therapy combined with chemotherapy reveals that at least 40 out of 49 listed studies have been evaluating chemotherapy drugs (ClinicalTrials.gov, 2021) (53). In summary, chemotherapeutic agents have an important role in MBC, and whether this approach could improve efficacy while reducing toxicity would be determined by ongoing clinical trials using targeted drugs in combination with these agents. The next frontier in MBC treatment is the combination of chemotherapy and targeted drugs.

### Cluster 3: Molecular mechanisms of MBC

Metastasis is a complex multi-step process (54). It was influenced by various molecular regulatory factors, such as gene expression, signaling pathways, epigenetics, and splicing variants. For example, E-cadherin is a cell adhesion molecule with a dual effect in breast cancer metastasis: on one hand, it inhibits the aggressiveness of cancer cells; on the other hand, it facilitates their survival in the blood and their proliferation in new organs (55). Another example is MBD2, a DNA methylation binding protein with two different splice variants MBD2a and MBD2c, which has opposite effects on breast cancer metastasis: MBD2a promotes tumor metastasis by enhancing FZD1 gene expression to activate the Wnt/β-catenin pathway and epithelial-mesenchymal transition (EMT); tumor metastasis is impeded by MBD2c by competitively inhibiting the effect of MBD2a on FZD1 gene (56).

In addition to the intrinsic properties of cancer cells, their surrounding microenvironment can also influence the metastasis of breast cancer cells. This microenvironment consists of various components, such as normal cells, extracellular matrix, blood vessels, immune system, and so on. They can promote or inhibit the proliferation and metastasis of cancer cells by providing them with nutrients, oxygen, signaling molecules and other factors (57). For instance, solid breast cancer tumors are usually characterized by hypoxia, which triggers the expression of hypoxia-inducible factors (HIFs) and activates a series of genes associated with tumor metastasis (58). Furthermore, a significant fraction of the tumor microenvironment is occupied by tumor-associated macrophages (TAMs), which can be divided into two subtypes: M1 and M2. The former subtype exhibits anti-tumor activity and secretes pro-inflammatory cytokines, oxidants and nitric oxide to eradicate cancer cells; the latter subtype displays pro-tumor activity and secretes anti-inflammatory cytokines, growth factors and angiogenic factors to facilitate the growth, migration and angiogenesis of cancer cells. TAMs typically present an M2-like phenotype in breast cancer, thus contributing to metastasis (59).

### Cluster 4: Liquid biopsy for MBC

Liquid biopsy is a diagnostic tool that minimally invades the body and allows the detection of biomarkers in blood or other bodily fluids for early detection and diagnosis of disease and monitoring (60), which is now a very promising method for monitoring the response to treatment and disease progression in MBC (61). Liquid biopsies involve the analysis of various biomolecules such as CTCs, which are cancer cells that break away from the primary tumor and enter the bloodstream, and ctDNA, which is the genetic material freed by dying tumor cells. Several studies have demonstrated that liquid biopsy can be used to predict the outcomes of MBC patients. For example, Pro. Cristofanilli collected and tested blood samples from nearly 2,500 MBC patients at 18 centers, including 533 women with newly discovered MBC. They found that an elevated number of CTCs predicts a poor prognosis (62). In addition, Pro. Shaw explored the characteristics of CTCs and ctDNA in a sample of 112 MBC patients and found that elevated ctDNA levels were significantly associated with shorter OS (63). Liquid biopsy can also be used to identify genetic mutations in the tumor that may influence treatment options, such as the presence of HER2 mutations that may make the tumor responsive to HER2-targeted therapies (64). Despite his many advantages, it is worth noting that liquid biopsy is not yet the standard diagnostic tool for MBC and further studies are required to confirm its usefulness and cost-effectiveness.

### Cluster 5: Survival and quality of life of MBC

Numerous factors influence the QoL and survival of individuals with MBC, such as the breast cancer subtype, the metastasis’ extent and location, the treatments’ availability and effectiveness, and the personal preferences and goals of care. Despite some advances in the management of MBC over the past two decades, triple-negative breast cancer remains poorly treated and its treatment options are limited. New agents, such as trastuzumab, pertuzumab, lapatinib, have demonstrated enhanced PFS and OS in clinical trials. OS denotes the duration from diagnosis to death, whereas PFS denotes the duration from treatment to disease progression or death. However, these endpoints may not adequately capture the full impact of MBC on QoL, which encompasses physical, emotional, social, and functional dimensions (65). Since PFS is often used as a surrogate endpoint for OS in clinical trials, several studies have investigated the relationship between PFS and QoL in patients with MBC. These studies have revealed that PFS may be correlated with QoL in some dimensions, such as symptom alleviation and functional status, but not in others, such as emotional well-being and personal goals. Furthermore, PFS may not account for the individual variation in QoL among MBC patients, as some patients may prioritize different dimensions of QoL more than others. Consequently, PFS alone may be insufficient to capture the patient perspective on treatment efficacy and benefit (65, 66). In summary, studies on QoL and survival in MBC patients over the past 20 years have highlighted the importance of incorporating the patient’s perspective as part of the design and implementation of clinical trials and health technology assessments.

### Cluster 6: Hormone receptor-positive MBC

Endocrine therapy mainly includes selective estrogen receptor modulators (SERM), and aromatase inhibitors (AI), which can impede estrogen stimulation of cancer cells through competitive binding to ER or through inhibition of estrogen synthesis (67). These agents have been the cornerstone of hormone receptor-positive MBC treatment for years. Fulvestrant, a selective estrogen receptor degrader (SERD), has been introduced into the clinic as a second-line treatment that can more potently inhibit the ER signaling pathway by inducing ER degradation. Fulvestrant exhibits superior efficacy to AIs in monotherapy or in combination with AIs (68). However, endocrine therapy alone is often inadequate to control the disease, as resistance mechanisms emerge over time (69). Consequently, researchers have devised new strategies to surmount endocrine resistance and defer disease progression.

Based on clinical trials, a number of molecularly targeted drugs have been approvable by the FDA for use in patients with hormone receptor-positive MBC. Everolimus, an inhibitor targeting mTOR, can improve the effectiveness of endocrine therapies for patients by inhibiting the PI3K/AKT/mTOR signaling pathway (70). A combination of everolimus and fulvestrant showed prolonged PFS in patients who failed endocrine therapy (71). Another class of molecular targeted drugs is cyclin-dependent kinase 4/6 (CDK4/6) inhibitors, which can block the binding of CDK4/6 with cyclin D1 or cyclin D3 and prevent the phosphorylation and inactivation of retinoblastoma (Rb) protein (72). Endocrine therapy combined with CDK4/6 inhibitors has become the first-line standard of care for hormone receptor-positive MBC (71). Other targeted agents, such as PARP inhibitors, may also be used for patients with PIK3CA or BRCA1/2 mutations (73), PARP inhibitors can induce synthetic lethality in cells with defective DNA repair mechanisms. Recently, SG, an antibody-drug conjugate targeting trophoblast surface antigen 2 (Trop-2) with SN-38 as the active payload, has been approved for the treatment of hormone receptor-positive MBC (74).

Moreover, several novel drugs are undergoing clinical trials, such as oral SERDs, PI3K/mTOR dual inhibitors, which can simultaneously inhibit both PI3K and mTOR and block the PI3K/AKT/mTOR signaling pathway more effectively. AKT inhibitors are also being studied, which can target the downstream effector of PI3K and prevent the activation of mTOR. Immunotherapy is another promising strategy, which can enhance the anti-tumor immune response by modulating the interaction between tumor cells and immune cells. However, there are still some unresolved issues, such as the optimal sequence of these novel regimens, the mechanisms of resistance to these drugs, the biomarkers that can predict the response or resistance to these drugs, etc.

The emergence of novel topics or trends can be detected by the analysis of keyword bursts. In the selected years, prior to 2010, the predominant burst keywords pertained to chemotherapy, clinical trials, and HER2-related terms, indicating that the treatment of MBC was predominantly oriented towards these aspects before the widespread adoption of molecular typing. Subsequent to 2010, particularly in recent years, burst keywords encompassed CDK4/6 inhibitors, circulating tumor cells, liquid biopsy, and so forth. These topics were also discussed in clusters 4 and 6. The above findings suggest that the diagnosis and treatment of hormone receptor-positive MBC has become a promising direction for MBC research.

To further investigate the recent trends and developments of MBC research in the past five years, we performed a co-citation analysis and a co-occurrence analysis of keywords for the publications from 2018 to 2022. A visual representation of the co-cited literature on MBC-related research during this period is displayed in **Supplementary Figure 2A**. The top ten literature in this field from 2018 to 2022, ranked by citation frequency and centrality, are summarized in **Supplementary Table 1**. We observed that, in contrast to **Table 5**, most of the literature were published after 2010. The paper with the highest citation frequency was a report on the phase III clinical trial based on PALOMA-1/TRIO-18 by Finn RS et al (75). This paper was the first to verify the efficacy and safety of CDK4/6 inhibitor Palbociclib in combination with endocrine therapy for hormone receptor-positive MBC patients, altering the first-line treatment standard for this subtype of MBC and offering a new effective and well-tolerated treatment option for these patients. The paper with the highest centrality was a prospective, multicenter study by Cristofanilli M et al., which corroborated that the level of circulating tumor cells is an independent prognostic indicator in patients with metastatic breast cancer, providing a simple, fast, non-invasive, and repeatable biomarker detection method for this patient population (76). These results indicate to some extent that the current research hotspots in the MBC field are concentrated on hormone receptor-positive MBC and liquid biopsy. Moreover, **Supplementary Figure 2B** shows a visual representation of the co-occurrence analysis of keywords for MBC-related research from 2018 to 2022. We found that it had similar clustering results to **Figure 6A**.

However, our study was not devoid of limitations. Firstly, we only retrieved publications from the WoS core collection. Consequently, this study did not incorporate relevant literature from other databases. Secondly, although the database is continuously updated, we only included publications from January 2002 to December 2022, which may entail the exclusion of some of the most recent research results. These may constitute some of the sources of bias in our study.

## Conclusion

In this paper, we synthesized the pertinent knowledge of MBC from a visual and bibliometric perspective, and attained a comprehensive and intuitive understanding of this topic. Simultaneously, we identified six research hotspots in the field of MBC and reviewed them. Among them, chemotherapy and HER2-positive MBC have a long and established research history. However, hormone receptor-positive MBC and liquid biopsy of MBC are the future research directions in this field. Moreover, further research on these issues will facilitate us to better elucidate the molecular mechanisms of MBC and guide its treatment.

## Data availability statement

The datasets presented in this study can be found in online repositories. The names of the repository/repositories and accession number(s) can be found in the article/**Supplementary Material**.

## Ethics statement

This study only used published or publicly available data. Ethical approval for each study included in the investigation can be found in the original publications (including informed consent from each participant).

## Author contributions 

SJ and YL conceived and designed the study. SJ and QM analyzed the data and wrote the first draft of the manuscript. FJ and YY completed the software operation, and XL and WS revised the manuscript. All authors contributed to the article and approved the submitted version.

## Supplementary material

Supplementary Figure 1
**(A)** MBC research of the binary matrix bicluster analysis of the visual mountain map. **(B)** Visualization matrix of binary matrix bicluster analysis for MBC correlation research.

Supplementary Figure 2
**(A)** Visualization of reference co-citation networks from 2018 to 2022. **(B)** Visualization of Keyword co-word analysis networks from 2018 to 2022.
