# Supplementary figures and images for: A bibliometric analysis of metastatic breast cancer: two-decade report (2002-2022)
Source: Front Oncol. 2023 Aug 24;13:1229222. doi: 10.3389/fonc.2023.1229222 (PMC10484517; doi:10.3389/fonc.2023.1229222)

A

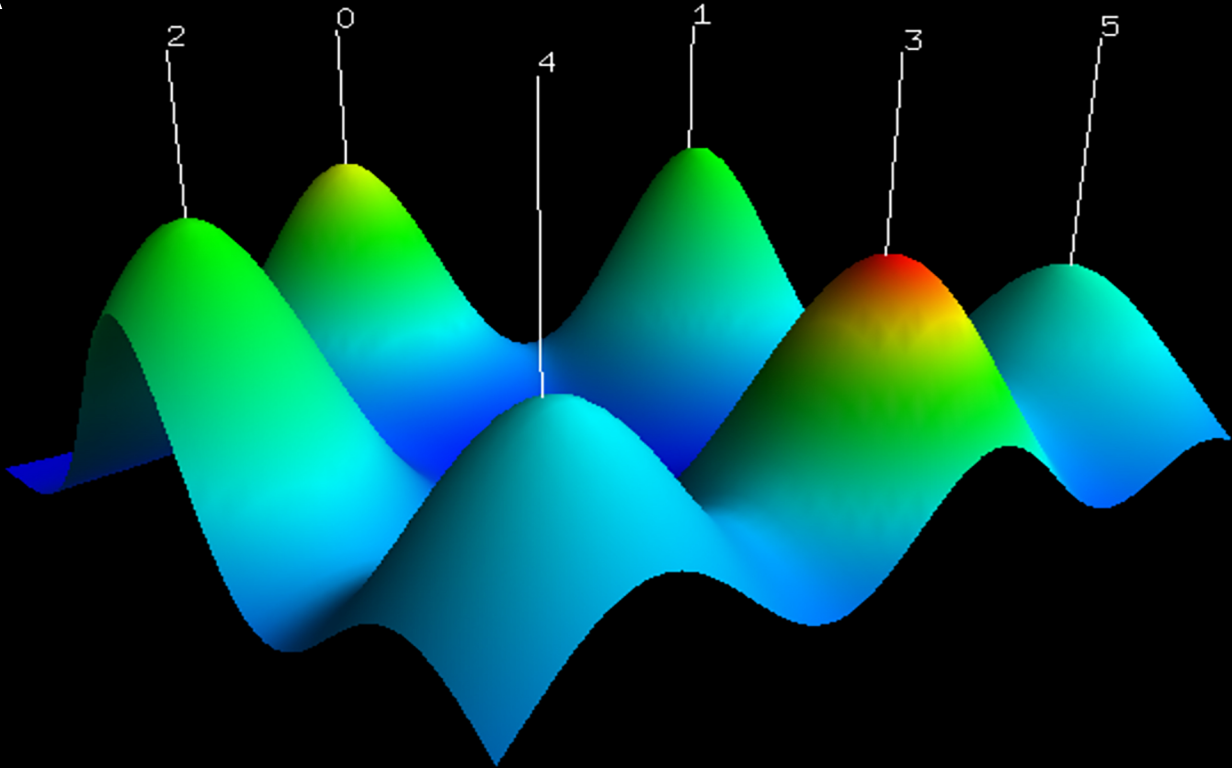

B

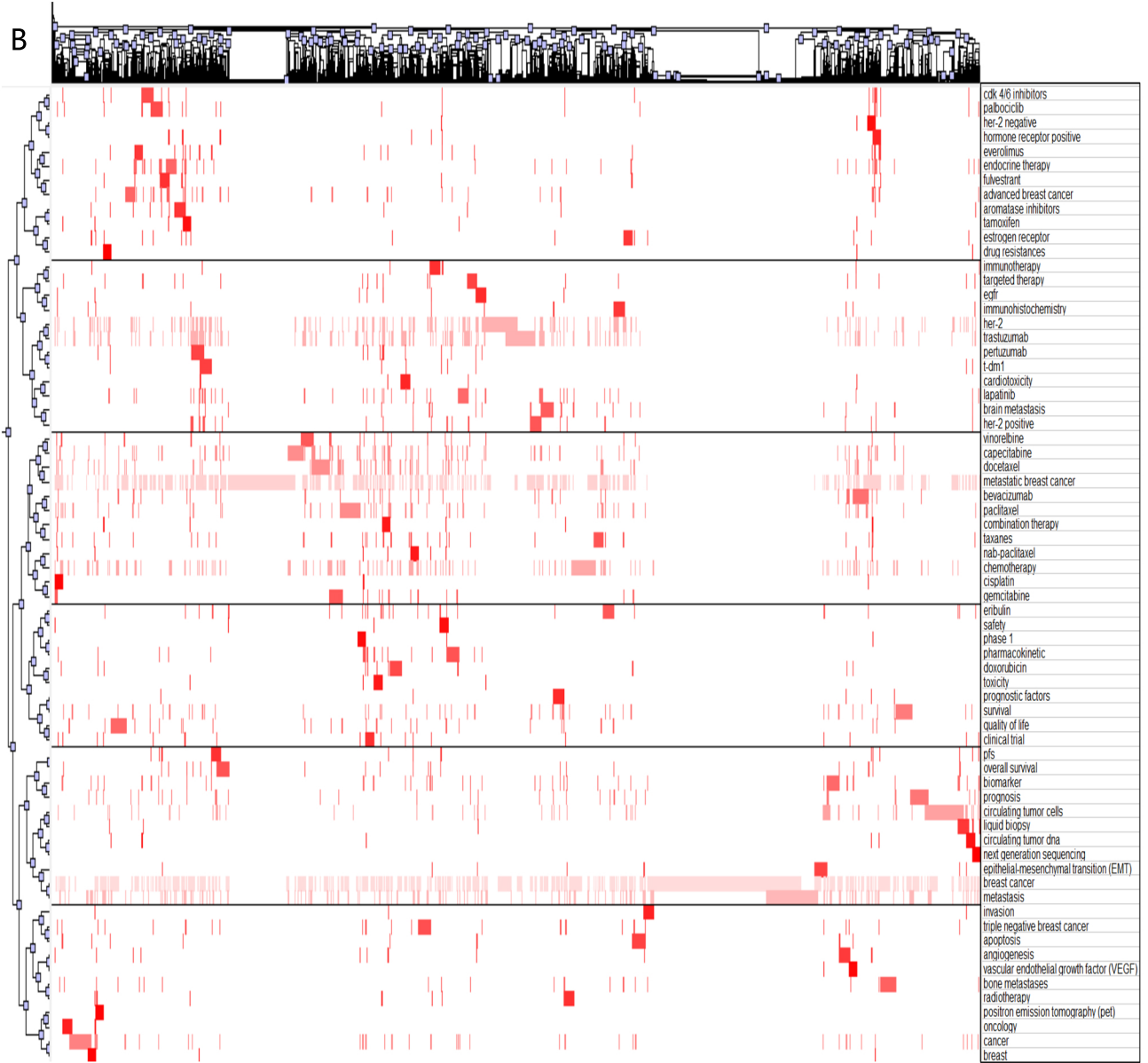

Supplement: Supplementary Figure 1 — (A) MBC research of the binary matrix bicluster analysis of the visual mountain map. (B) Visualization matrix of binary matrix bicluster analysis for MBC correlation research. [file Image_1.pdf]

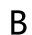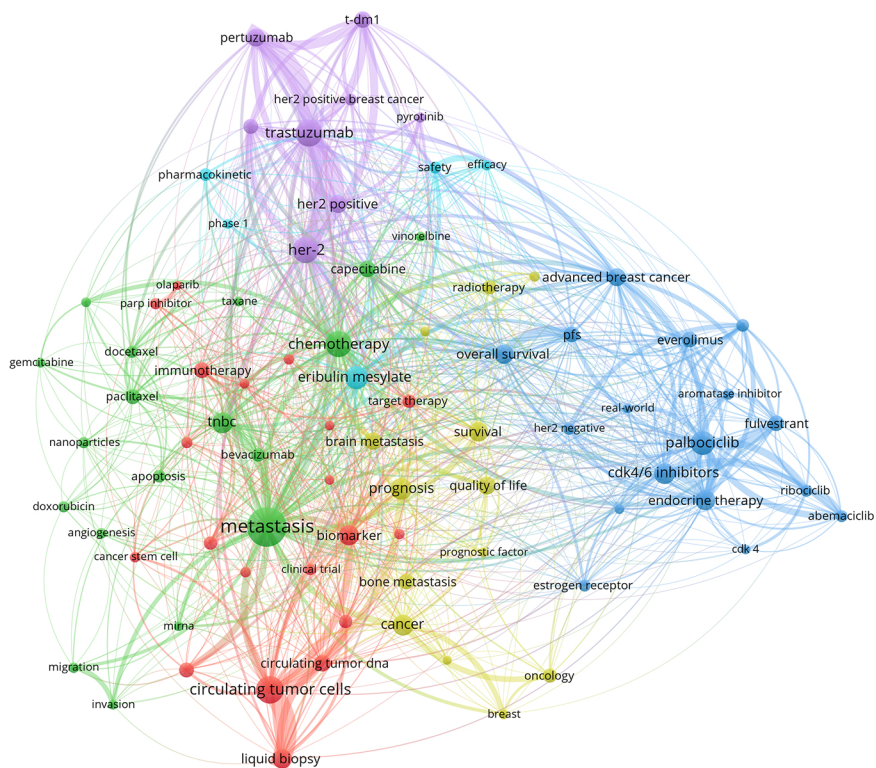

Supplement: Supplementary Figure 2 — (A) Visualization of reference co-citation networks from 2018 to 2022. (B) Visualization of Keyword co-word analysis networks from 2018 to 2022. [file Image_2.pdf]
