# Supplementary material for: A bibliometric analysis of metastatic breast cancer: two-decade report (2002-2022)
Source: Front Oncol. 2023 Aug 24;13:1229222. doi: 10.3389/fonc.2023.1229222 (PMC10484517; doi:10.3389/fonc.2023.1229222)
Supplement: Supplementary file 4 [file Table_1.docx]

| Supplymentary Table 1. The top 10 co-cited references based on citation counts and centrality in 2018-2022. | | | | | | |
| --- | --- | --- | --- | --- | --- | --- |
| Rank | Citation Counts | References | DOI | Centrality | References | DOI |
| 1 | 329 | Finn RS, 2016, NEW ENGL J MED, V375, P1925 | 10.1056/NEJMoa1607303 | 0.10 | Cristofanilli M, 2004, NEW ENGL J MED, V351, P781 | 10.1056/NEJMoa040766 |
| 2 | 305 | Eisenhauer EA, 2009, EUR J CANCER, V45, P228 | 10.1016/j.ejca.2008.10.026 | 0.10 | Robertson JFR, 2016, LANCET, V388, P2997 | 10.1016/S0140-6736(16)32389-3 |
| 3 | 269 | Bray F, 2018, CA-CANCER J CLIN, V68, P394 | 10.3322/caac.21492 | 0.08 | Kaufman PA, 2015, J CLIN ONCOL, V33, P594 | 10.1200/JCO.2013.52.4892 |
| 4 | 262 | Slamon DJ, 2001, NEW ENGL J MED, V344, P783 | 10.1056/NEJM200103153441101 | 0.07 | Murtaza M, 2013, NATURE, V497, P108 | 10.1038/nature12065 |
| 5 | 247 | Cristofanilli M, 2016, LANCET ONCOL, V17, P425 | 10.1016/S1470-2045(15)00613-0 | 0.07 | Hyman DM, 2018, NATURE, V554, P189 | 10.1038/nature25475 |
| 6 | 205 | Verma S, 2012, NEW ENGL J MED, V367, P1783 | 10.1056/NEJMoa1209124 | 0.07 | Ma F, 2017, J CLIN ONCOL, V35, P3105 | 10.1200/JCO.2016.69.6179 |
| 7 | 205 | Swain SM, 2015, NEW ENGL J MED, V372, P724 | 10.1056/NEJMoa1413513 | 0.07 | Nik-Zainal S, 2016, NATURE, V534, P47 | 10.1038/nature17676 |
| 8 | 205 | Cardoso F, 2018, ANN ONCOL, V29, P1634 | 10.1093/annonc/mdy192 | 0.07 | Robson ME, 2019, ANN ONCOL, V30, P558 | 10.1093/annonc/mdz012 |
| 9 | 182 | Koboldt DC, 2012, NATURE, V490, P61 | 10.1038/nature11412 | 0.06 | Finn RS, 2016, NEW ENGL J MED, V375, P1925 | 10.1056/NEJMoa1607303 |
| 10 | 179 | Goetz MP, 2017, J CLIN ONCOL, V35, P3638 | 10.1200/JCO.2017.75.6155 | 0.06 | Blackwell KL, 2012, J CLIN ONCOL, V30, P2585 | 10.1200/JCO.2011.35.6725 |
